# The gastric disease of Napoleon Bonaparte: brief report for the bicentenary of Napoleon’s death on St. Helena in 1821

**DOI:** 10.1007/s00428-021-03061-1

**Published:** 2021-03-04

**Authors:** Alessandro Lugli, Fatima Carneiro, Heather Dawson, Jean-François Fléjou, Richard Kirsch, Rachel S. van der Post, Michael Vieth, Magali Svrcek

**Affiliations:** 1 Institute of Pathology, University of Bern, Murtenstrasse 31, 3008 Bern, Switzerland; 2 Centro Hospitalar Universitario de São João/Medical Faculty of Porto, Porto, Portugal; 3 Institute of Molecular Pathology and Immunology of the University of Porto/i3S - Instituto de Investigação e Inovação em Saúde, Universidade do Porto, Porto, Portugal; 4 Département de Pathologie, Cerbapath, Paris, France; 5 Pathology and Laboratory Medicine, Mount Sinai Hospital, University of Toronto, Toronto, Ontario Canada; 6 Department of Pathology, Radboud Institute for Molecular Life Sciences, Radboud University Medical Centre, Nijmegen, the Netherlands; 7 Institute of Pathology, Klinikum Bayreuth Friedrich-Alexander University Erlangen-Nuremberg, Erlangen, Germany; 8 Sorbonne Université, AP-HP, Hôpital Saint-Antoine, Service d’Anatomie et cytologie pathologiques, Paris, France

**Keywords:** Napoleon, Bicentenary, Gastritis, Gastric cancer, Autopsy report

## Abstract

After the defeat at the battle of Waterloo on June 18, 1815, Napoleon Bonaparte was sent into exile to the Island of St. Helena where he died 6 years later on May 5, 1821. One day after his death, Napoleon’s personal physician, Dr. Francesco Antommarchi, performed the autopsy in the presence of Napoleon’s exile companions and the British medical doctors. Two hundred years later, mysteries still surround the cause of his death and different hypotheses have been postulated in the medical and historical literature. The main reasons seem to be the presence of several autopsy reports, their interpretation and perhaps the greed for thrill and mystery. Therefore, for the bicentenary of Napoleon’s death, an international consortium of gastrointestinal pathologists assembled to analyse Napoleon’s autopsy reports based on the level of medical evidence and to investigate if the autopsy reports really do not allow a final statement.

## Introduction

Napoleon Bonaparte died on May 5, 1821, on the Atlantic Island of St. Helena. According to the autopsy reports of Napoleon’s personal physician, Dr. Francesco Antommarchi (1789–1838), and the British medical doctors present at the autopsy, the pathological findings of Napoleon’s stomach highly suggest a malignant gastric neoplasia. Nevertheless, even 200 years later, mysteries surround the Emperor’s death. The presence of an elevated arsenic concentration in Napoleon’s hair raised the hypothesis of arsenic poisoning [1]. Other suggested reasons for Napoleon’s death, causing quite a stir, are iatrogenic drug–induced intoxication [2] and, more recently, chronic gastritis associated with anaemia due to gastrointestinal bleeding [3]. The diagnosis of gastric cancer is often challenged based on the following arguments: first, the gastric lesion is not malignant; second, if the lesion is malignant, then only at an early stage and ultimately not the cause of death; third, Napoleon’s clinical history is not compatible with gastric cancer; fourth, Dr. Antommarchi’s second autopsy report published in his memoirs in 1825 is a plagiarism. For the bicentenary of Napoleon’s death, an international consortium of gastrointestinal pathologists reanalysed the different autopsy reports and investigated on their medical reliability with the aim to make a final conclusion on Napoleon’s cause of death.

## The level of medical evidence of Napoleon’s autopsy reports

Napoleon’s autopsy was performed on May 6, 1821, at 2 p.m. at Longwood House on the Island of St. Helena in the presence of a French and a British delegation. Napoleon’s French retinue included Dr. Francesco Antommarchi, Napoleon’s personal physician, Count Montholon, General Bertrand, the valet St Denis and Marchand, the butler Pierron and the priest Abbe Vignali. The British committee was represented by seven MDs, namely Archibald Arnott, Thomas Shortt, Charles Mitchell, Francis Burton, Matthew Livingstone supported by Walter Henry and George Henry Rutledge and also by Sir Thomas Reade representing Sir Hudson Lowe, Governor of the Island, Major Charles Hamilton and the Duty Officer at Longwood House William Crokat [4].

In this brief review, we therefore only investigated the sources originating from the people present at the autopsy and we further stratified the reports into three medical evidence levels (strong, moderate and weak):

### Strong medical evidence

The first autopsy report of Francesco Antommarchi and the British autopsy report are written by medical doctors at or just after the autopsy. They show medical knowledge and a precise description of the pathological findings. Antommarchi’s first autopsy report was included in Montholon’s and Marchand’s memoirs [5, 6], while the official British report is preserved in the Lowe Papers at the British Library in London [7].

### Moderate medical evidence

Dr. Walter Henry, Assistant Surgeon, took notes during the autopsy in 1821 which were part of a letter he wrote to Sir Hudson Lowe, Governor of the Island of St. Helena, in 1823 [7]. Despite the similarity of the pathological findings described in the reports of 1821, the delay of 2 years may imply some potential impreciseness.

### Weak medical evidence

General Henri Gatien Bertrand was Napoleon’s exile companion and his diary was published in Paris in the twentieth century [4]. Bertrand is not a medical doctor and the short description of the pathological findings does not add any further information. Louis-Etienne Saint-Denis was Napoleon’s valet. Despite his presence at the autopsy, he did not take any notes, but published his memoirs several years later including a short description of Napoleon’s stomach at the autopsy: “There was a perforation in the stomach and around this perforation many little holes as made by lead shots of a pistol” [8]. Compared to the autopsy reports of 1821, the description is incomplete and from a medical point of view not at a professional level.

Antommarchi published in 1825 “Les Derniers Moments de Napoleon”, including a second autopsy report with additional findings not originally described [9]. In 2006, this report was found to have striking similarities with an article published in the French medical journal Archives Générales de Médecine in 1823 and since then should be considered a plagiarism and was therefore excluded from the present review [9].

A short overview of Napoleon’s different autopsy reports is summarized in Table 1.

**Table 1:** Overview of Napoleon’s different autopsy reports

Author	Source	Medical evidence
Francesco Antommarchi (1789–1838)	Official French autopsy report published in: Montholon’s memoirs entitled “Récits de la captivité de l’empereur Napoléon à Sainte-Hélène” (Paris, 1847) Mémoires de Marchand, premier valet de chambre et éxécuteur testamentaire de l’empereur Napoléon (Paris, 1952–1955)	*Strong* Antommarchi was Napoleon’s personal physician and anatomist. The autopsy report was signed on May 8, 1821
Thomas Shortt (1788–1843) Archibald Arnott (1772–1855) Charles Mitchell (1783–1856) Francis Burton (1784–1828) Matthew Livingstone (1773–1821)	Official British autopsy report entitled ““Report of Appearances on Dissection of the Body of Napoleon Bonaparte”. Preserved in the Lowe Papers at the British Library, London	*Strong* Five British MDs who were present at the autopsy and signed the report on May 6, 1821
Walter Henry (1791–1860)	Letter to Sir Hudson Lowe	*Moderate* Walter Henry was Assistant Surgeon and took notes during Napoleon’s autopsy. The postmortal findings were included in a letter written two years after the autopsy on September 12, 1823.
Henri Gatien Bertrand (1773–1844)	Cahiers de Sainte-Hélène	*Weak* Bertrand was Grand Maréchal du Palais. His diary was published between in Paris in the twentieth century.
Louis-Etienne Saint-Denis (1788–1856)	Souvenirs du mameluck Ali sur l’empereur Napoléon	*Weak* Louis-Etienne Saint-Denis (Ali) was Napoleon’s valet. He never took notes, but wrote his memoirs later in Sens, France between 1827 and 1856. His souvenirs were published in Paris in 1926.

## Interpretation of the autopsy reports of 1821 signed by Dr. Antommarchi and the British doctors

Some summarized excerpts with focus on the pathological findings in Napoleon’s stomach are presented in Table 2 [6, 7]. Their interpretation allows the following five statements:

1. The cardiac extremity for a small space near the termination of the oesophagus was the only part appearing in a healthy state.
2. The stomach was nearly filled with a large quantity of black liquid of disagreeable odour resembling coffee grounds.
3. The internal surface of the stomach to nearly its whole extent, extending from the cardiac orifice to about an inch from the pylorus, was a mass of cancerous disease/scirrhous portions advancing to cancer/extensive cancerous ulcer.
4. The ulcerated surface of the stomach was considerably swollen and indurated.
5. Presence of a perforated ulcer (diameter: 6–7mm) which was located one inch from the pylorus. The ulcer was covered by strong adhesions located between the stomach and the liver.

**Table 2:** Summarized extracts from the first autopsy report of Francesco Antommarchi in comparison to the British autopsy report with focus on the pathological findings in Napoleon’s stomach [5–7]

Antommarchi, May 8, 1821	British doctors, May 6, 1821
L’adhérence de la face concave du lobe gauche du foie formait un trou du diamètre d’environ trois “lignes” dans la face antérieure de l’estomac, près de son extrémité droite. The adhesion of the concave surface of the left hepatic lobe was covering a hole (three “lignes”* in diameter) located in the anterior gastric surface near to its right extremity. L’estomac derrière était rempli en partie d’une substance liquide, noirâtre, d’une odeur piquante et désagréable. *The stomach was partially filled with a liquid and black substance of disagreeable odour.* Un ulcère cancéreux fort étendu occupait spécialement la partie supérieure de la face interne de l’estomac, et s’étendait de l’orifice du cardia jusqu’à environ un pouce du pilorum. *There was an extensive cancerous ulcer especially in the upper part of the internal gastric surface that extended from the cardia to about one inch from the pylorus*. Sur les bords de cet ulcère, vers le pilorum, le trou ci-dessus désigné, produit par corrosion ulcéreuse des parois de l’estomac. *Towards the pylorus, on this ulcer’s edge, the above mentioned hole produced by ulcerous corrosion of the gastric coat.* Les parois ulcéreuses de l’estomac étaient considérablement gonflées et endurcies. *The gastric surface was indurated and extremely swollen.* Entre l’ulcère et le pilorum, et contigus à l’ulcère, gonflement et dureté squirreuse de la largeur de quelques “lignes”, qui occupaient circulairement l’extrémité droite de l’estomac. *Between the pylorus and the ulcer, just next to the ulcer, there was a scirrhous hardness, several “lignes” in size, forming a circular mass at the right gastric extremity.*	The omentum was found remarkably fat and the stomach was found the seat of extensive disease. Strong adhesions connected the whole superior surface, particularly about the pyloric extremity to the concave surface of the left lobe of the liver. An ulcer which penetrated the coats of the stomach was discovered one inch from the pylorus sufficient to allow the passage of the little finger. The internal surface of the stomach to nearly its whole extent was a mass of cancerous disease or scirrhous portions advancing to cancer, this was particularly noticed near the pylorus. The cardiac extremity for a small space near the termination of the oesophagus was the only part appearing in a healthy state. The stomach was found nearly filled with a large quantity of fluid resembling coffee grounds.

The summary of Dr. Antommarchi’s autopsy report is exceptionally presented in French to avoid any misunderstandings and bias due to translation. For completeness, the English translation is also available at the bottom of each paragraph (*1ligne = approximately 2.256mm)

## Clinico-pathological aspects of Napoleon’s final disease

For medical doctors specialized in gastrointestinal diseases, these statements are very suggestive for the diagnosis of an advanced malignant gastric neoplasia (gastric carcinoma and/or gastric lymphoma) associated with a covered perforated ulcer and upper gastrointestinal bleeding.

Nevertheless, since Napoleon’s autopsy, the diagnosis of gastric cancer was questioned several times in the medico-historical literature. In 1961, an elevated arsenic concentration in Napoleon’s hair taken after his death suggested arsenic poisoning [1]. This hypothesis was finally dismissed by a study published in 2008 showing an elevated arsenic content in the hair of Napoleon during his childhood as well as in the hairs of Napoleon’s son and Joséphine [10]. This study along with others excluded an arsenic poisoning with criminal intent [3, 7, 11, 12]. In 2012, a medico-historical book challenges gastric cancer again and a chronic gastritis associated with gastrointestinal bleeding and anaemia was suggested as Napoleon’s cause of death instead [3]. The following clinico-pathological aspects clearly show why this hypothesis stays on an extremely shaky ground:

Based on the WHO classification of digestive tumours (5th edition 2019), common symptoms of gastric cancer, especially in advanced stages, include asthenia, indigestion, vomiting, weight loss, dysphagia, early satisfaction of appetite and anaemia [13]. According to historical, sources Napoleon showed quite several of these symptoms, especially in the last few months of his life [4–6, 8, 14]. Additionally, the anaemia may be simply tumour-related.

On May 3, 1821, 2 days before his death, Napoleon was given Calomel (mercurous chloride) by his doctors leading to the hypothesis of an iatrogenic drug–induced cause of death. Napoleon was already tachycardic before May 3 [4] which supports along with the postmortal findings his advanced malignant gastric neoplasia being the cause for the gastric bleeding and Calomel just a trigger. Napoleon’s health status was decreasing since October 1820 which is in line with cancer progression and makes an “unnatural” component [12] unlikely.

The macroscopic description in the two original autopsy reports does not favour chronic gastritis at all. In previous publications, the macroscopy of Napoleon’s stomach was compared with several pictures of non-treated gastritis and gastric cancer [11] [14]. None of the gastritis pictures were in the least comparable to the description in Napoleon’s autopsy reports in contrast to the macroscopic aspect of gastric cancer Borrmann subtype III. The description of Saint-Denis in his memoirs stating “There was a perforation in the stomach and around this perforation many little holes as made by lead shots of a pistol” [8], may be well-intentioned, but clearly incomplete and not medically sound.

There is a strong evidence for an association between tumour size and tumour stage in gastric cancer [11, 14]. Indeed, Napoleon’s gastric lesion is associated with an advanced gastric cancer even according to the autopsy report of 1821 by Antommarchi and the one signed by the British doctors, respectively [11, 14]. Having excluded Antommarchi’s autopsy report of 1825, the lack of evident metastases in the autopsy reports from 1821 is still not surprising as often loco-regional lymph node metastases are only detected microscopically. Since histological confirmation of the cancer is not available, we cannot be sure of the histological subtype. However, the metastatic pattern of both intestinal and diffuse type gastric cancer is often associated with (distant) lymph nodes and peritoneal metastases (with or without ascites) which may be easily missed during the autopsy. Nevertheless, according to Siddharta Mukherjee’s book “The emperor of all maladies. A biography of cancer”, there is strong evidence that cancer was a known entity before 1821 [15].

The co-existence of malignant gastric neoplasia (carcinoma and/or lymphoma) and gastric ulcer with perforation may suggests a *Helicobacter pylori* gastritis associated carcinogenesis [13].

## Conclusion

The autopsy reports signed just after Napoleon’s autopsy on May 6 and May 8 by the British doctors and Francesco Antommarchi show strong medical evidence and allow a final diagnosis: advanced malignant gastric neoplasia associated with upper gastrointestinal bleeding as cause of death. Napoleon lost his final battle against an enemy who even in our days is unfortunately still strong: cancer, emperor of all maladies.

**Fig. 1:**
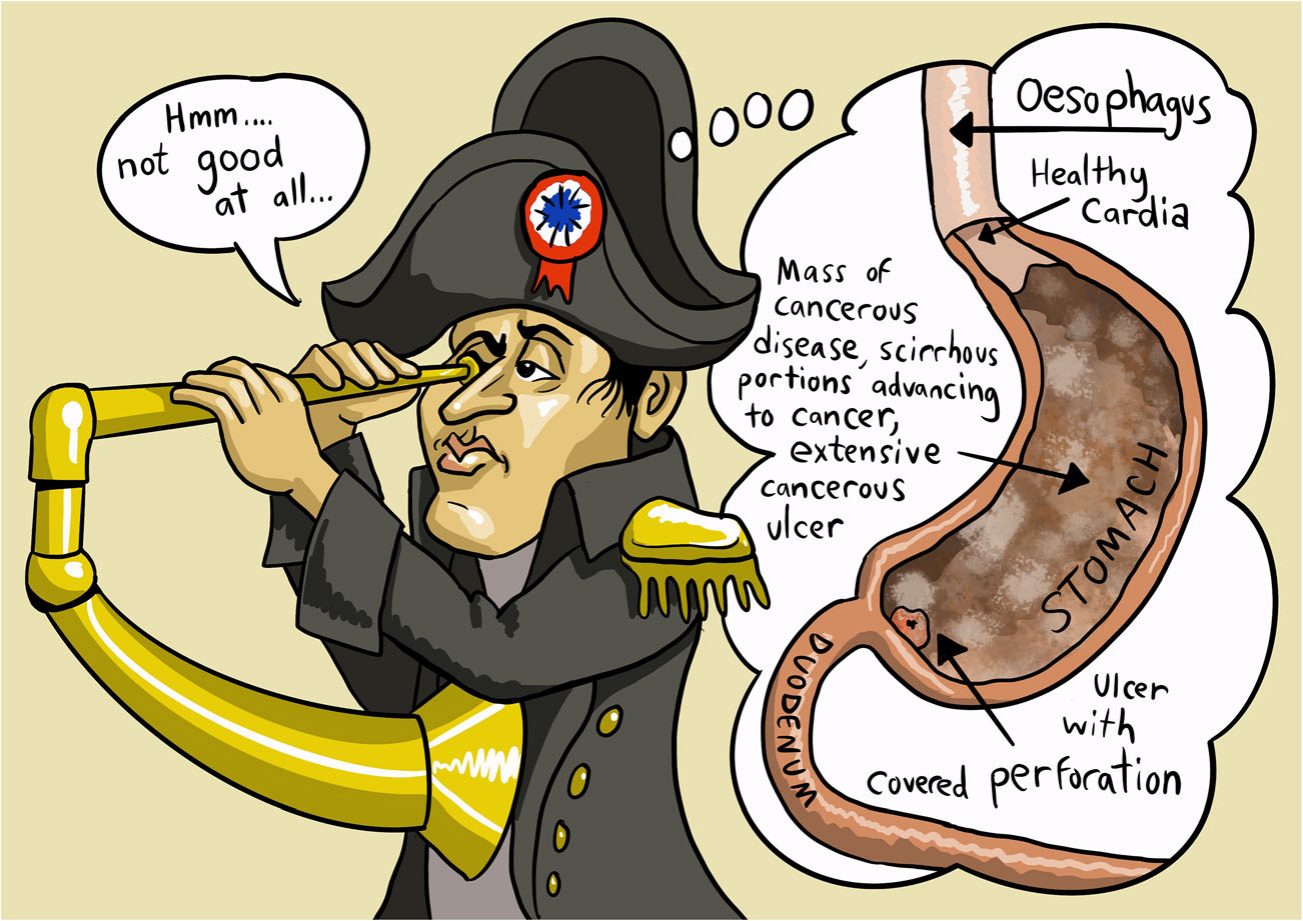
Morphologic visualization of Napoleon’s gastric lesion based on the autopsy reports of Francesco Antommarchi (May 8, 1821) and the British doctors, Thomas Shortt, Arch Arnott, Charles Mitchell, Francis Burton and Matthew Livingstone (May 6, 1821). Created by David Levine based on an idea of Alessandro Lugli
